# Accuracy augmentation of body composition measurement by bioelectrical impedance analyzer in elderly population

**DOI:** 10.1097/MD.0000000000019103

**Published:** 2020-02-14

**Authors:** Wen-Hui Fang, Jie-Ru Yang, Chih-Ying Lin, Po-Jen Hsiao, Ming-Yu Tu, Chien-Fu Chen, Dung-Jang Tsai, Wen Su, Guo-Shu Huang, Hung Chang, Sui-Lung Su

**Affiliations:** a Department of Family and Community Medicine, Tri-Service General Hospital; b School of Public Health, National Defense Medical Center, Taipei; c Department of Orthopedics; d Department of Internal Medicine, Taoyuan Armed Forces General Hospital, Taoyuan; e Department of Orthopedics, Kaohsiung Armed Forces General Hospital Gangshan Branch, Kaohsiung; f Department of Orthopedics, Taichung Armed Forces General Hospital, Taichung; g Graduate Institute of Life Sciences, National Defense Medical Center; h Department of Nursing; i Department of Radiology, Tri-Service General Hospital; j Department of Physiology and Biophysics, National Defense Medical Center; k Division of Thoracic Surgery, Tri-Service General Hospital, Taipei, Taiwan, ROC.

**Keywords:** bioelectrical impedance analyzer, body composition, dual X-ray absorptiometry, predictive model

## Abstract

Bioelectrical impedance analysis (BIA) is currently the most commonly used method in clinical practice to measure body composition. However, the bioelectrical impedance analyzer is not designed according to different countries, races, and elderly populations. Because different races may have different body compositions, a prediction model for the elderly population in Taiwan should be developed to avoid population bias, thereby improving the accuracy of community evaluation surveys.

Dual energy X-ray absorptiometry (DXA) was used as a standard method for comparison, and impedance analysis was used for the development of a highly accurate predictive model that is suitable for assessing the body composition of elderly people.

This study employed a cross-sectional design and recruited 438 elderly people who were undergoing health examinations at the health management center in the Tri-Service General Hospital as study subjects. Basic demographic variables and impedance analysis values were used in four predictive models, namely, linear regression, random forest, support vector machine (SVM), and eXtreme Gradient Boosting (XGBoost) models, to predict DXA body composition. The data from 354 study subjects were used to develop the predictive model, while the data from 84 study subjects were used to validate the accuracy of the predictive model.

The body composition of elderly people as estimated by InBody 720 was highly correlated with that estimated by DXA. The correlation coefficient between InBody 720 and DXA for muscle mass was 0.969, and that for fat mass was 0.935. Consistency analysis results showed that InBody 720 tends to underestimate muscle mass and fat mass. A comparison of the accuracy of the linear regression, random forest, SVM, and XGBoost models showed that the linear regression has the highest accuracy. The correlation coefficient between the new model and DXA for muscle mass and fat mass were 0.977 and 0.978, respectively.

The new predictive model can be used to monitor the nutrition status of elderly people and identify people with sarcopenia in the community.

## Abstract 2

Supplemental Digital Content is available in the text

## 1 Introduction

Body composition has a large impact on health and disease.^[1]^ In elderly people, changes in body composition are often associated with sarcopenia, malnutrition, disability, obesity, cardiovascular diseases, and others.^[2]^ Muscle senescence and atrophy are important markers for aging in the human body. Aging is accompanied by the loss of skeletal muscle mass and strength. As people age, their muscle mass gradually decreases, and this decline accelerates after the age of 50 years.^[3,4]^ However, malnutrition can also result in a decline in fat and muscle mass, thereby affecting muscle function, motor skills, and health status and even leading to death.^[5–7]^ Therefore, accurate assessments of fat mass and muscle mass in body composition measurements are important for assessing the health and nutrition status of elderly people.^[8]^

Dual energy X-ray absorptiometry (DXA) is regarded in many studies as the gold standard for body composition measurements in clinical experiments.^[9–11]^ DXA is a safe and noninvasive method that not only has high precision, reproducibility, and accuracy but also can be used for systemic and local body composition assessments.^[12]^ However, there are many limitations in the use of DXA, including the high equipment costs, difficulty associated with the operation and maintenance of the equipment, poor portability, radioactivity, and inability to be used for periodic examinations.^[13]^ DXA is most commonly used by medical institutions for clinical diagnoses and assessments. It is not convenient for use in community evaluations and examinations and is thus not widely used.

Bioelectrical impedance analysis (BIA) is based on the fact that different tissues in the body have different amounts of water content and cell membrane characteristics, which produce different levels of resistance and reactance to currents of different frequencies. With different models and formulas, these impedance values can be used to deduce body composition.^[14]^ Due to the presence of reactance in the cell membrane, the cell membrane has high capacitive reactance under low frequencies, and currents generally only pass through extracellular fluid and do not pass through the cell membrane. As the current frequency increases, the capacitive reactance of the cell membrane gradually decreases, and the proportion of currents that pass through intracellular routes subsequently increase. There may be differences in the fat distribution and body density between individuals of different races, which can affect the accuracy of the formulas.^[14,15]^ BIA is currently a widely used method for evaluating body composition in epidemiological studies conducted in the community. BIA is also regarded as an alternative method for evaluating body composition in elderly people.^[16]^ BIA is easy to execute, cheap, fast, and noninvasive, and it has been proven to have good reliability in some studies.^[17]^ However, BIA measurements may vary due to differences between individuals in factors such as race, ethnicity, device, age, and sex.^[14]^ Therefore, if a predictive model between BIA and DXA measurement methods for elderly people in Taiwan can be developed, it will provide a rapid, convenient, and precise method of tracking the body composition status of elderly people.

The bioelectrical impedance analyzer was not designed according to individuals in different countries and of different races and is widely used in various age groups. However, body composition does change with age, and the bioelectrical impedance analyzer was not designed for elderly populations; in addition, individuals of different races may have different body composition characteristics. Currently, the bioelectrical impedance analyzers that are commonly used in Taiwan are made in Europe, the USA, Japan, and South Korea. These manufacturers mostly derived equations based on the body composition status of residents in their respective countries. Many previous studies have also noted that most models were designed based on Caucasians, African Americans, Hispanic Americans, and Native Americans.^[18–20]^ Currently, there is no consistency between the body composition values measured by DXA and BIA in elderly people aged 65 years and above in Taiwan. Moreover, the body composition distribution in Taiwan is also different from those of other countries. Therefore, the aim of this study is to use DXA and BIA to develop a predictive model that is suitable for estimating the body composition of elderly people in Taiwan.

## 2 Materials and methods

### 2.1 Subjects

**Figure 1:**
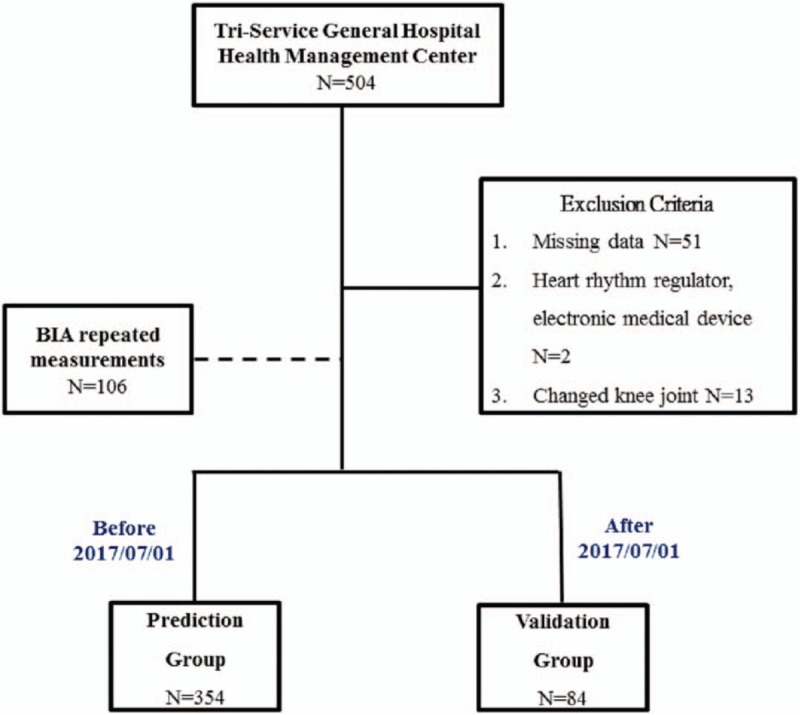
Process of screening the study subjects.

This study employed a cross-sectional research design, and elderly people who were undergoing health examinations at the Tri-Service General Hospital were recruited between March 2017 and August 2017. The recruiting method involved voluntary participation. All the subjects did not consume alcohol for at least 48 h, perform vigorous exercise for at least 12 h, or consume a meal or drink for at least 12 h prior to the examination. The subjects were also required to empty their bladder immediately before the BIA measurement. Interviewers who were trained in administering standardized interviews explained the purpose of the study to the study subjects and obtained consent from the subjects who signed the IRB consent form (TSGH- 2–102–05–028), which was approved by the institutional review board of Tri-service General Hospital before the interviews were carried out. Structured questionnaires were used to obtain basic demographic data from the study subjects. Next, BIA measurements were performed in the morning to obtain BIA body composition data. Afterwards, the patients underwent systemic DXA scans to obtain DXA body composition data. These two measurements were completed within 30 min at the health examination center. The exclusion criteria were patients with missing data, a BMI >50 kg/m^2^, pacemakers, or electronic medical devices and patients who underwent amputation or knee replacement surgery. Figure 1 shows the screening process: 504 elderly people were selected as study subjects. After patients were excluded based on the exclusion criteria, a total of 438 elderly people were included in the study. Two repeated InBody 720 measurements of 106 study subjects were taken. Then, the data of the 438 elderly people were divided into 2 groups. The data from the 354 elderly people who participated in the study before 1 July were included in the prediction group, while the data from the 84 elderly people who participated after 1 July were included in the validation group. The data from the prediction group were used as training data, while the data from the validation group were used as test data.

This study was approved by the institutional review board of the Tri-Service General Hospital (TSGH), a medical teaching hospital of the National Defense Medical Center in Taipei, and the Taiwanese volunteers signed the informed consent form after the investigators explained the study.

### 2.2 Methods

#### 2.2.1 Anthropometric measurements

Body weight was measured to the nearest 0.1 kg using a standard scale as subjects wore light clothing and no shoes; barefoot standing height was measured to the nearest 0.1 cm by using a wall-mounted stadiometer; and BMI was calculated as the weight in kilograms divided by the height in meters squared.

#### 2.2.2 Dual-energy X-ray absorptiometry (DXA)

As the reference method, dual-energy X-ray absorptiometry (DXA) was used for measuring whole and regional body composition, including fat mass, lean mass, and bone mineral content. The subjects were dressed in cotton robes without metal attachments. They were placed in a supine position in the center of the scanning field with their palms facing downwards, their arms lying away from their body, either straight or at a slight angle, their feet in a neutral position, and their face facing upwards with their chin in a neutral position. The scan took approximately 180 s to complete, and the dose of radiation per individual was 0.01 mGy (1.0 mrad). The composition of the four limbs was measured in grams by the DXA software.

#### 2.2.3 Bioelectrical impedance analysis

Bioelectrical impedance analysis (BIA) was carried out with a body composition analyzer (InBody 720; Biospace). A tetra-polar eight-point tactile electrode system was used. The system separately measured the impedance of the participants’ right arm, left arm, trunk, right leg, and left leg at six different frequencies (1, 5, 50, 250, 500, and 1000 kHz) for each body segment. In accordance with the manufacturer's guidelines, the participants wiped the bottom of their feet with a proprietary electrolyte tissue before standing on the electrodes embedded in the scale platform of the respective analyzers. The participants were instructed to stand upright and to grasp the handles of the analyzer, thereby providing contact with a total of eight electrodes (2 for each foot and hand).

#### 2.2.4 Statistical analysis

The basic demographic variables were expressed as quantities and percentages. Continuous variables were expressed as means ± SDs and were assessed by Student's *t* test. The intrarater reliability and intraclass correlation coefficient (ICC) of the repeated InBody 720 measurements for the same subject were measured. The level of linear correlation between BIA and DXA was tested using Pearson correlation (*r*). The Bland-Altman plot was used to compare the consistency of the continuous data between the BIA and DXA clinical measurement methods. The steps for the predictive model were as follows: First, samples were selected, and 354 elderly people who underwent health examinations before 1 July were included in the prediction group. The data obtained for the variables were used for feature engineering. The feature engineering method used in this study utilizes relevant knowledge for data domains, where the height squared was divided by the impedance values of the various body parts. Next, the variables were selected. The random forest method was used to measure the importance of each variable, which was then allocated into layers (depths) according to importance. Next, the average number of layers for each variable was arranged in sequence, and the top 20, 30, 40, and 50 most important variables were included in the model. Finally, the selected variables were used to construct a model, and linear regression, random forest, support vector machine (SVM) and eXtreme Gradient Boosting (XGBoost) were used to construct predictive models for the body composition of each body part. In addition, the data from the validation group were used to validate the accuracy of the model. The Pearson correlation coefficient was used to identify the variable and predictive method with the best prediction.

## 3 Results

### 3.1 Distribution of the basic information of the study subjects

Table 1 shows the distribution of the basic demographic data. There were 179 males (40.9%) and 259 females (59.1%). There were no statistically significant differences in the distribution of sex (prediction group: males: 41.2%; validation group: males 39.3%), age (prediction group: 73.32 ± 6.83 years; validation group: 72.95 ± 6.85 years), height (prediction group: 158.70 ± 8.19 cm; validation group: 158.12 ± 8.34 cm), weight (prediction group: 60.94 ± 10.61 kg; validation group: 60.24 ± 11.47 kg), or body mass index (prediction group: 24.12 ± 3.28; validation group: 23.98 ± 3.53) between the prediction and validation groups.

**Table 1:**
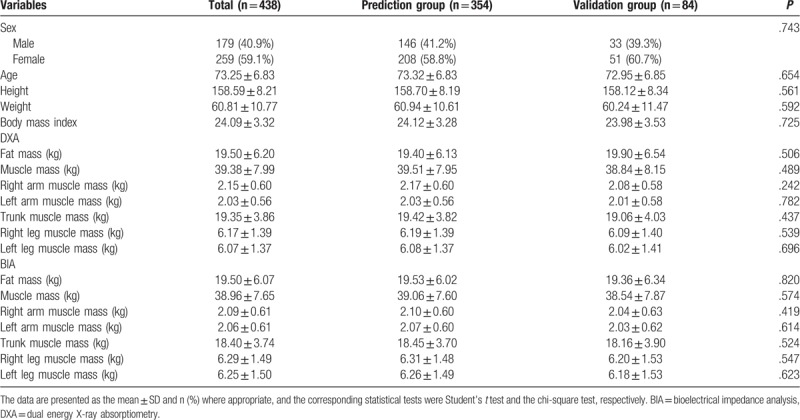
Distribution of the basic demographic data of the study subjects.

### 3.2 Intragroup consistency analysis in repeated BIA measurements

Table 2 shows the intragroup concordance correlation coefficient and correlation coefficient for repeated BIA measurements. Two repeated InBody 720 measurements were taken from 106 subjects by the same operator following the same steps. The intragroup correlation coefficients for fat mass, muscle mass, left and right arm muscle mass, trunk muscle mass, and left and right leg muscle mass were all 0.99, and all correlations showed statistical significance (*P* < .001).

**Table 2:**
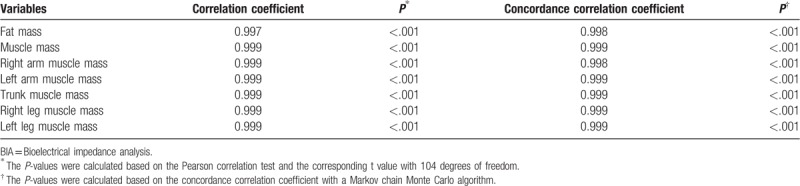
Intra-group consistency analysis for the repeated BIA measurements.

### 3.3 Correlation between BIA and DXA body composition

Table 3 shows the correlation between BIA (InBody 720) and DXA body composition: The concordance correlation coefficient between BIA (InBody 720) and DXA for fat mass, muscle mass, right arm muscle mass, left arm muscle mass, trunk muscle mass, right leg muscle mass, and left leg muscle mass were 0.952, 0.967, 0.933, 0.936, 0.899, 0.930, and 0.919, respectively, and all correlations showed statistical significance.

**Table 3:**
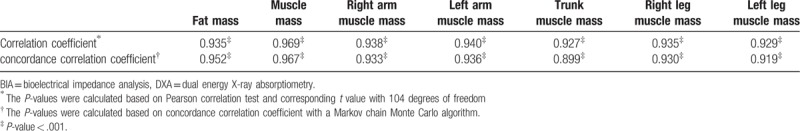
Correlation between BIA and DXA in body composition.

### 3.4 Consistency of BIA and DXA body composition

Figure 2   A–G shows the Bland–Altman difference plots comparing the consistency between the BIA (InBody 720) and DXA methods of measuring body composition. The solid line shows the mean difference between BIA (InBody 720) and DXA body composition, while the dotted line represents the 95% confidence interval. From the graph, we can see that BIA (InBody 720) tends to underestimate fat mass (estimated bias of 0.0019 and 95% limits of agreement −3.71, 3.71) and muscle mass (estimated bias of 0.42 and 95% limits of agreement −3.44, 4.28).

**Figure 2:**
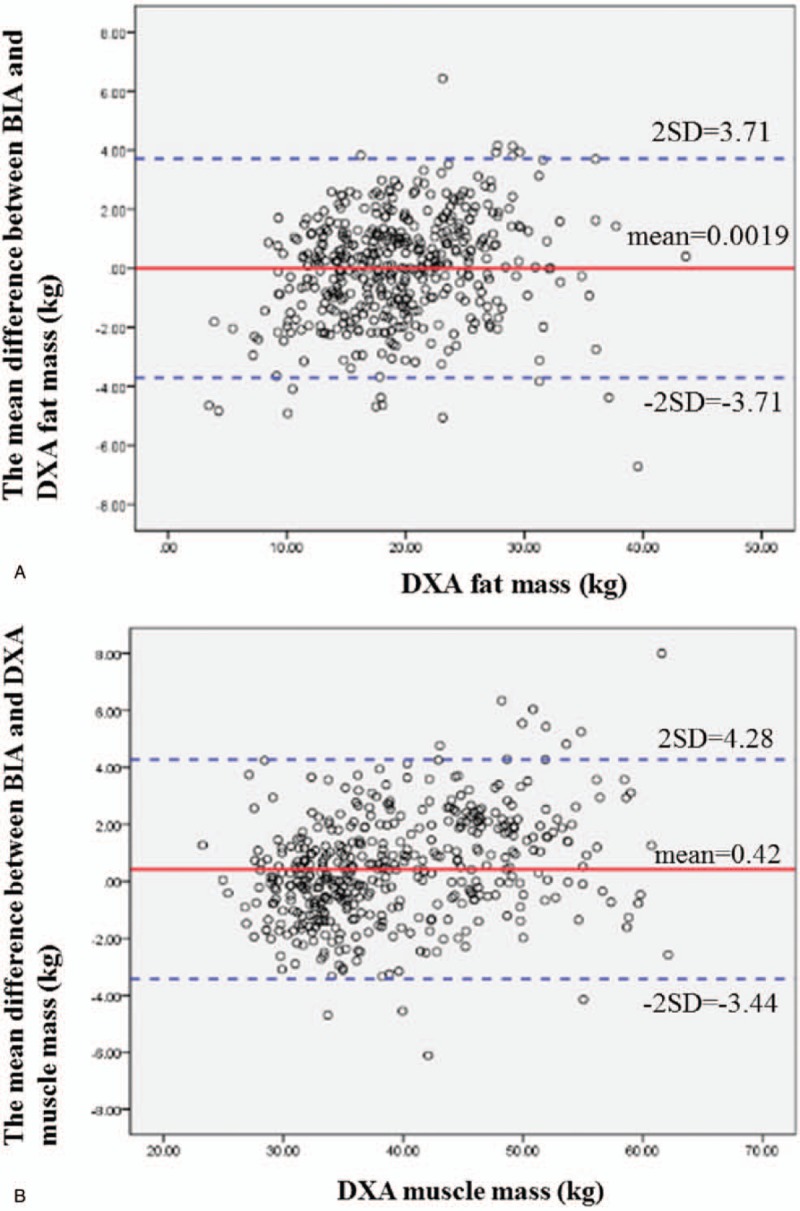
(A) Bland–Altman difference plot for BIA and DXA adipose-derived mass. The mean difference in fat mass between BIA and DXA was 0.0019, and the confidence interval for the two standard deviations was −3.71 to 3.71. The Bland–Altman consistency analysis results showed that BIA tends to underestimate fat mass. However, overall, only <5% of the differences in fat mass between DXA and BIA exceeded ±2 standard deviations, which conforms to the 95% confidence level. Therefore, the fat masses estimated by DXA and BIA are consistent. (B) Bland–Altman difference plot for BIA and DXA muscle mass. The mean difference in muscle mass between BIA and DXA was 0.42, and the confidence interval for the two standard deviations was −3.44 to 4.28. The Bland–Altman consistency analysis results showed that BIA tends to underestimate muscle mass. However, overall, only <5% of the differences in muscle mass between DXA and BIA exceeded ±2 standard deviations, which conforms to the 95% confidence level. Therefore, the muscle masses estimated by DXA and BIA are consistent. (C) Bland–Altman difference plot for BIA and DXA right arm muscle mass. The mean difference in right arm muscle mass between BIA and DXA was 0.061, and the confidence interval for the two standard deviations was −0.36 to 0.48. The Bland–Altman consistency analysis results showed that BIA tends to underestimate the right arm muscle mass. However, overall, only <5% of the differences in muscle mass between DXA and BIA exceeded ±2 standard deviations, which conforms to the 95% confidence level. Therefore, the muscle masses estimated by DXA and BIA are consistent. (D) Bland–Altman difference plot for BIA and DXA left arm muscle mass. The mean difference in left arm muscle mass between BIA and DXA was −0.032, and the confidence interval for the two standard deviations was −0.44 to 0.37. The Bland–Altman consistency analysis results showed that BIA tends to overestimate the left arm muscle mass. However, overall, only <5% of the differences in muscle mass between DXA and BIA exceeded ±2 standard deviations, which conforms to the 95% confidence level. Therefore, the muscle masses estimated by DXA and BIA are consistent. (E) Bland–Altman difference plot for BIA and DXA trunk muscle mass. The mean difference in trunk muscle mass between BIA and DXA was 0.95, and the confidence interval for the two standard deviations was −1.89 to 3.8. The Bland–Altman consistency analysis results showed that BIA tends to underestimate the trunk muscle mass. However, overall, only <5% of the differences in muscle mass between DXA and BIA exceeded ± 2 standard deviations, which conforms to the 95% confidence level. Therefore, the muscle masses estimated by DXA and BIA are consistent. (F) Bland–Altman difference plot for BIA and DXA right leg muscle mass. The mean difference in right leg muscle mass between BIA and DXA was −0.12, and the confidence interval for the two standard deviations was −1.15 to 0.92. The Bland–Altman consistency analysis results showed that BIA tends to overestimate the right leg muscle mass. However, overall, only <5% of the differences in muscle mass between DXA and BIA exceeded ±2 standard deviations, which conforms to the 95% confidence level. Therefore, the muscle masses estimated by DXA and BIA are consistent. (G) Bland–Altman difference plot for BIA and DXA left leg muscle mass. The mean difference in left leg muscle mass between BIA and DXA was −0.18, and the confidence interval for the two standard deviations was −1.26 to 0.91. The mean difference in right leg muscle mass between BIA and DXA was −0.12, and the confidence interval for the two standard deviations was −1.15 to 0.92. The Bland–Altman consistency analysis results showed that BIA tends to overestimate the left leg muscle mass. However, overall, only <5% of the differences in muscle mass between DXA and BIA exceeded ±2 standard deviations, which conforms to the 95% confidence level. Therefore, the muscle masses estimated by DXA and BIA are consistent.

**Figure 2 (Continued):**
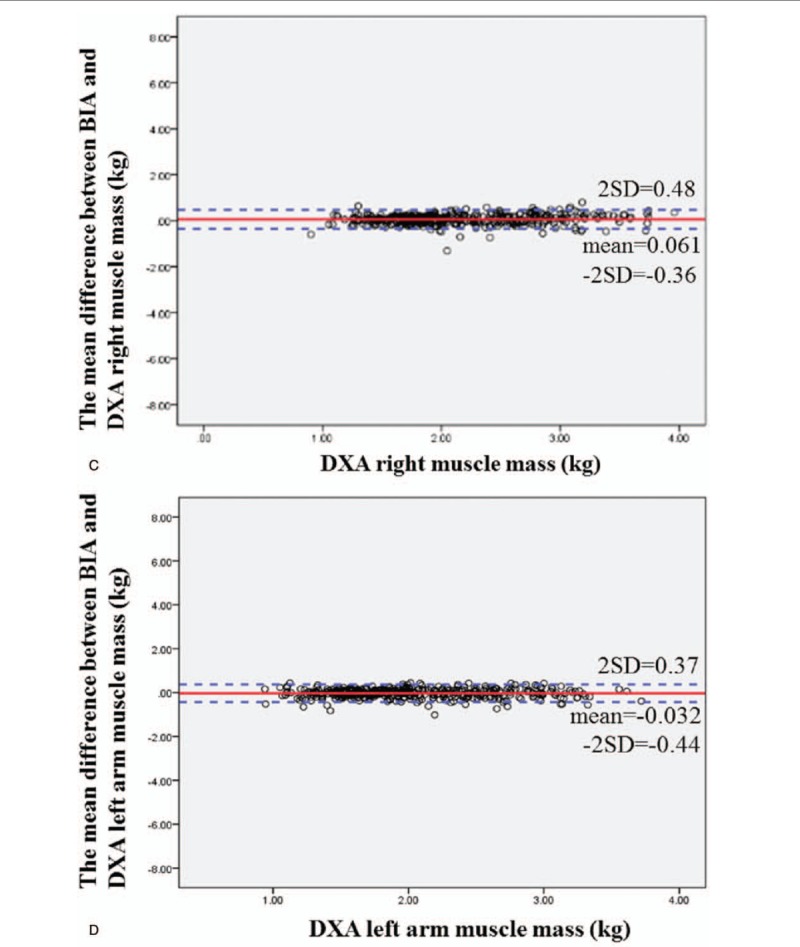
(A) Bland–Altman difference plot for BIA and DXA adipose-derived mass. The mean difference in fat mass between BIA and DXA was 0.0019, and the confidence interval for the two standard deviations was −3.71 to 3.71. The Bland–Altman consistency analysis results showed that BIA tends to underestimate fat mass. However, overall, only <5% of the differences in fat mass between DXA and BIA exceeded ±2 standard deviations, which conforms to the 95% confidence level. Therefore, the fat masses estimated by DXA and BIA are consistent. (B) Bland–Altman difference plot for BIA and DXA muscle mass. The mean difference in muscle mass between BIA and DXA was 0.42, and the confidence interval for the two standard deviations was −3.44 to 4.28. The Bland–Altman consistency analysis results showed that BIA tends to underestimate muscle mass. However, overall, only <5% of the differences in muscle mass between DXA and BIA exceeded ±2 standard deviations, which conforms to the 95% confidence level. Therefore, the muscle masses estimated by DXA and BIA are consistent. (C) Bland–Altman difference plot for BIA and DXA right arm muscle mass. The mean difference in right arm muscle mass between BIA and DXA was 0.061, and the confidence interval for the two standard deviations was −0.36 to 0.48. The Bland–Altman consistency analysis results showed that BIA tends to underestimate the right arm muscle mass. However, overall, only <5% of the differences in muscle mass between DXA and BIA exceeded ±2 standard deviations, which conforms to the 95% confidence level. Therefore, the muscle masses estimated by DXA and BIA are consistent. (D) Bland–Altman difference plot for BIA and DXA left arm muscle mass. The mean difference in left arm muscle mass between BIA and DXA was −0.032, and the confidence interval for the two standard deviations was −0.44 to 0.37. The Bland–Altman consistency analysis results showed that BIA tends to overestimate the left arm muscle mass. However, overall, only <5% of the differences in muscle mass between DXA and BIA exceeded ±2 standard deviations, which conforms to the 95% confidence level. Therefore, the muscle masses estimated by DXA and BIA are consistent. (E) Bland–Altman difference plot for BIA and DXA trunk muscle mass. The mean difference in trunk muscle mass between BIA and DXA was 0.95, and the confidence interval for the two standard deviations was −1.89 to 3.8. The Bland–Altman consistency analysis results showed that BIA tends to underestimate the trunk muscle mass. However, overall, only <5% of the differences in muscle mass between DXA and BIA exceeded ± 2 standard deviations, which conforms to the 95% confidence level. Therefore, the muscle masses estimated by DXA and BIA are consistent. (F) Bland–Altman difference plot for BIA and DXA right leg muscle mass. The mean difference in right leg muscle mass between BIA and DXA was −0.12, and the confidence interval for the two standard deviations was −1.15 to 0.92. The Bland–Altman consistency analysis results showed that BIA tends to overestimate the right leg muscle mass. However, overall, only <5% of the differences in muscle mass between DXA and BIA exceeded ±2 standard deviations, which conforms to the 95% confidence level. Therefore, the muscle masses estimated by DXA and BIA are consistent. (G) Bland–Altman difference plot for BIA and DXA left leg muscle mass. The mean difference in left leg muscle mass between BIA and DXA was −0.18, and the confidence interval for the two standard deviations was −1.26 to 0.91. The mean difference in right leg muscle mass between BIA and DXA was −0.12, and the confidence interval for the two standard deviations was −1.15 to 0.92. The Bland–Altman consistency analysis results showed that BIA tends to overestimate the left leg muscle mass. However, overall, only <5% of the differences in muscle mass between DXA and BIA exceeded ±2 standard deviations, which conforms to the 95% confidence level. Therefore, the muscle masses estimated by DXA and BIA are consistent.

**Figure 2 (Continued):**
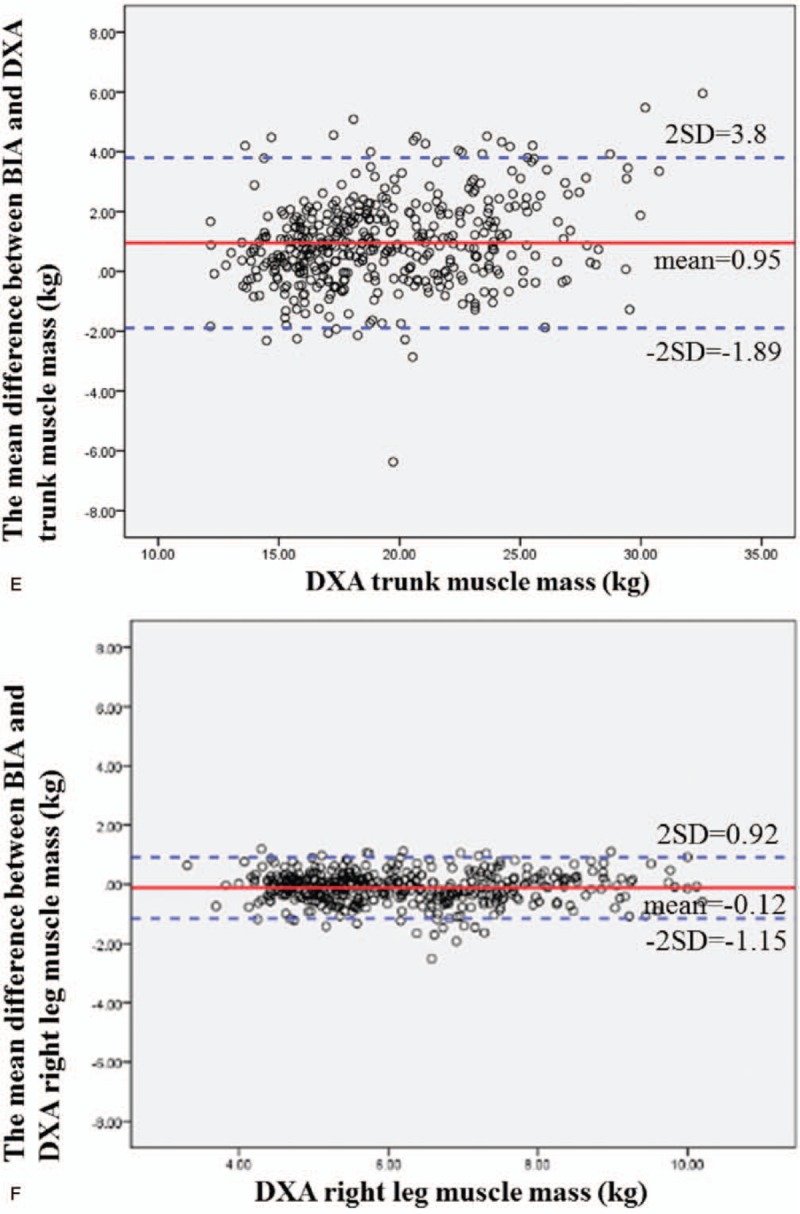
(A) Bland–Altman difference plot for BIA and DXA adipose-derived mass. The mean difference in fat mass between BIA and DXA was 0.0019, and the confidence interval for the two standard deviations was −3.71 to 3.71. The Bland–Altman consistency analysis results showed that BIA tends to underestimate fat mass. However, overall, only <5% of the differences in fat mass between DXA and BIA exceeded ±2 standard deviations, which conforms to the 95% confidence level. Therefore, the fat masses estimated by DXA and BIA are consistent. (B) Bland–Altman difference plot for BIA and DXA muscle mass. The mean difference in muscle mass between BIA and DXA was 0.42, and the confidence interval for the two standard deviations was −3.44 to 4.28. The Bland–Altman consistency analysis results showed that BIA tends to underestimate muscle mass. However, overall, only <5% of the differences in muscle mass between DXA and BIA exceeded ±2 standard deviations, which conforms to the 95% confidence level. Therefore, the muscle masses estimated by DXA and BIA are consistent. (C) Bland–Altman difference plot for BIA and DXA right arm muscle mass. The mean difference in right arm muscle mass between BIA and DXA was 0.061, and the confidence interval for the two standard deviations was −0.36 to 0.48. The Bland–Altman consistency analysis results showed that BIA tends to underestimate the right arm muscle mass. However, overall, only <5% of the differences in muscle mass between DXA and BIA exceeded ±2 standard deviations, which conforms to the 95% confidence level. Therefore, the muscle masses estimated by DXA and BIA are consistent. (D) Bland–Altman difference plot for BIA and DXA left arm muscle mass. The mean difference in left arm muscle mass between BIA and DXA was −0.032, and the confidence interval for the two standard deviations was −0.44 to 0.37. The Bland–Altman consistency analysis results showed that BIA tends to overestimate the left arm muscle mass. However, overall, only <5% of the differences in muscle mass between DXA and BIA exceeded ±2 standard deviations, which conforms to the 95% confidence level. Therefore, the muscle masses estimated by DXA and BIA are consistent. (E) Bland–Altman difference plot for BIA and DXA trunk muscle mass. The mean difference in trunk muscle mass between BIA and DXA was 0.95, and the confidence interval for the two standard deviations was −1.89 to 3.8. The Bland–Altman consistency analysis results showed that BIA tends to underestimate the trunk muscle mass. However, overall, only <5% of the differences in muscle mass between DXA and BIA exceeded ± 2 standard deviations, which conforms to the 95% confidence level. Therefore, the muscle masses estimated by DXA and BIA are consistent. (F) Bland–Altman difference plot for BIA and DXA right leg muscle mass. The mean difference in right leg muscle mass between BIA and DXA was −0.12, and the confidence interval for the two standard deviations was −1.15 to 0.92. The Bland–Altman consistency analysis results showed that BIA tends to overestimate the right leg muscle mass. However, overall, only <5% of the differences in muscle mass between DXA and BIA exceeded ±2 standard deviations, which conforms to the 95% confidence level. Therefore, the muscle masses estimated by DXA and BIA are consistent. (G) Bland–Altman difference plot for BIA and DXA left leg muscle mass. The mean difference in left leg muscle mass between BIA and DXA was −0.18, and the confidence interval for the two standard deviations was −1.26 to 0.91. The mean difference in right leg muscle mass between BIA and DXA was −0.12, and the confidence interval for the two standard deviations was −1.15 to 0.92. The Bland–Altman consistency analysis results showed that BIA tends to overestimate the left leg muscle mass. However, overall, only <5% of the differences in muscle mass between DXA and BIA exceeded ±2 standard deviations, which conforms to the 95% confidence level. Therefore, the muscle masses estimated by DXA and BIA are consistent.

**Figure 2 (Continued):**
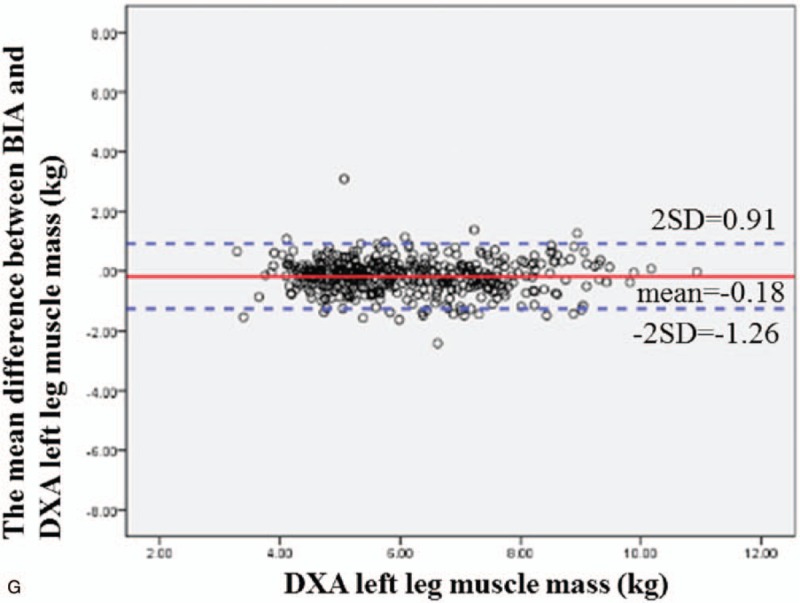
(A) Bland–Altman difference plot for BIA and DXA adipose-derived mass. The mean difference in fat mass between BIA and DXA was 0.0019, and the confidence interval for the two standard deviations was −3.71 to 3.71. The Bland–Altman consistency analysis results showed that BIA tends to underestimate fat mass. However, overall, only <5% of the differences in fat mass between DXA and BIA exceeded ±2 standard deviations, which conforms to the 95% confidence level. Therefore, the fat masses estimated by DXA and BIA are consistent. (B) Bland–Altman difference plot for BIA and DXA muscle mass. The mean difference in muscle mass between BIA and DXA was 0.42, and the confidence interval for the two standard deviations was −3.44 to 4.28. The Bland–Altman consistency analysis results showed that BIA tends to underestimate muscle mass. However, overall, only <5% of the differences in muscle mass between DXA and BIA exceeded ±2 standard deviations, which conforms to the 95% confidence level. Therefore, the muscle masses estimated by DXA and BIA are consistent. (C) Bland–Altman difference plot for BIA and DXA right arm muscle mass. The mean difference in right arm muscle mass between BIA and DXA was 0.061, and the confidence interval for the two standard deviations was −0.36 to 0.48. The Bland–Altman consistency analysis results showed that BIA tends to underestimate the right arm muscle mass. However, overall, only <5% of the differences in muscle mass between DXA and BIA exceeded ±2 standard deviations, which conforms to the 95% confidence level. Therefore, the muscle masses estimated by DXA and BIA are consistent. (D) Bland–Altman difference plot for BIA and DXA left arm muscle mass. The mean difference in left arm muscle mass between BIA and DXA was −0.032, and the confidence interval for the two standard deviations was −0.44 to 0.37. The Bland–Altman consistency analysis results showed that BIA tends to overestimate the left arm muscle mass. However, overall, only <5% of the differences in muscle mass between DXA and BIA exceeded ±2 standard deviations, which conforms to the 95% confidence level. Therefore, the muscle masses estimated by DXA and BIA are consistent. (E) Bland–Altman difference plot for BIA and DXA trunk muscle mass. The mean difference in trunk muscle mass between BIA and DXA was 0.95, and the confidence interval for the two standard deviations was −1.89 to 3.8. The Bland–Altman consistency analysis results showed that BIA tends to underestimate the trunk muscle mass. However, overall, only <5% of the differences in muscle mass between DXA and BIA exceeded ± 2 standard deviations, which conforms to the 95% confidence level. Therefore, the muscle masses estimated by DXA and BIA are consistent. (F) Bland–Altman difference plot for BIA and DXA right leg muscle mass. The mean difference in right leg muscle mass between BIA and DXA was −0.12, and the confidence interval for the two standard deviations was −1.15 to 0.92. The Bland–Altman consistency analysis results showed that BIA tends to overestimate the right leg muscle mass. However, overall, only <5% of the differences in muscle mass between DXA and BIA exceeded ±2 standard deviations, which conforms to the 95% confidence level. Therefore, the muscle masses estimated by DXA and BIA are consistent. (G) Bland–Altman difference plot for BIA and DXA left leg muscle mass. The mean difference in left leg muscle mass between BIA and DXA was −0.18, and the confidence interval for the two standard deviations was −1.26 to 0.91. The mean difference in right leg muscle mass between BIA and DXA was −0.12, and the confidence interval for the two standard deviations was −1.15 to 0.92. The Bland–Altman consistency analysis results showed that BIA tends to overestimate the left leg muscle mass. However, overall, only <5% of the differences in muscle mass between DXA and BIA exceeded ±2 standard deviations, which conforms to the 95% confidence level. Therefore, the muscle masses estimated by DXA and BIA are consistent.

### 3.5 Evaluation markers for predictive models of body composition in elderly people

Table 4 shows the optimal correlation coefficient and variables for body composition that were predicted by the different models. Overall, among the 4 predictive models, linear regression had the highest accuracy in predicting each variable for body composition, which was higher than the initial measurements from InBody 720. Therefore, this paper uses linear regression to analyze, develop, and validate new predictive models for the prediction of the body composition of elderly populations in Taiwan.

**Table 4:**
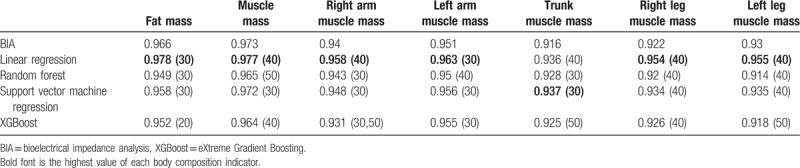
Optimal correlation coefficient and variables for the body composition predicted by different models.

### 3.6 Accuracy comparison between DXA, BIA, and the new prediction model

Table 5 shows DXA as a standard for comparison, and the BIA and new predictive model (regression) were used to evaluate the kappa value, sensitivity, and specificity of the skeletal muscle mass indices of the four limbs in the validation group (sarcopenia in Asians was defined as skeletal muscle mass indices of the four limbs that were lower than the standard value or matched the normal standard value: males: ≤7.0 kg/m^2^; females: ≤5.4 kg/m^2^). In addition, we also evaluated the kappa value, sensitivity, and specificity for the fat percentage in lean, normal, and overweight subjects (males-lean: <15%, normal: 15%–25%, overweight: >35%; females-lean: <25%, normal: 25%–33%, overweight: >33%). When the new predictive model was compared with BIA, the kappa value and sensitivity of the new predictive model in evaluating the skeletal muscle mass index of the four limbs were higher than the values for BIA. The kappa value for fat percentage for the new predictive model was also higher than that for BIA. When the lean category was compared with the normal and overweight categories, the sensitivity of the new predictive model was also found to be higher than that of BIA.

**Table 5:**
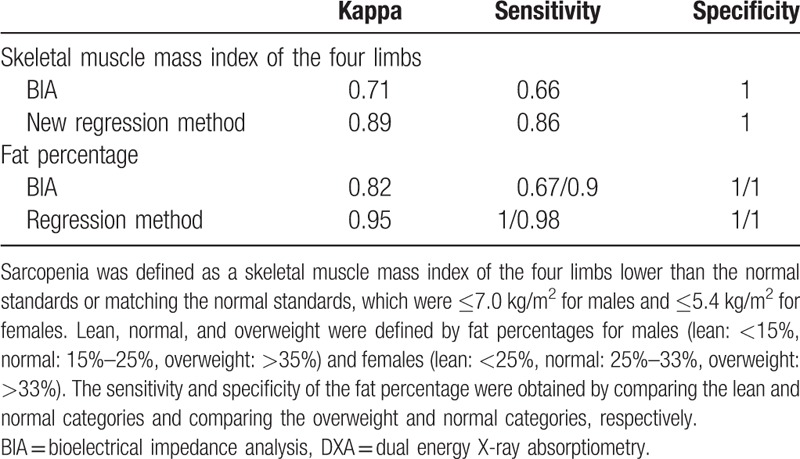
Accuracy comparison between DXA, BIA, and the new prediction model.

## 4 Discussion

The results of this study showed that the intragroup reliability of the repeated InBody 720 measurements taken by the same operator following the same steps was 0.99, and this result was statistically significant (*P* < .001). This result was consistent with those of other studies. Mally et al studied the reliability of BIA (Tanita BC-418) used for repeated muscle mass and fat mass measurements in elderly people in Europe. Their results showed that there were no significant differences between the first and second measurements for muscle mass and fat mass and that the two test results were highly correlated.^[21]^

The results of this study showed that the that InBody 720 estimation of muscle mass in elderly people is highly correlated with DXA measurements (*r* = 0.967). The Bland–Altman consistency analysis results showed that InBody 720 tends to underestimate muscle mass, but the estimations were still consistent overall. Kim et al studied 551 community-dwelling elderly subjects in Japan. Their study results proved that BIA (InBody 720) shows consistency in estimating whole body muscle mass. However, BIA (InBody 720) was similarly found to underestimate muscle mass during the validation process.^[22]^ In contrast, a Finnish study showed opposite results: Sillanpaa et al evaluated 882 adults aged 18 to 88 years and found that BIA (InBody 720) overestimated whole body muscle mass.^[23]^ The possible reason for the differences in the study results may be the differences in body structure between the individuals in different age groups and races and the differences in the sample size.

With regard to the measurement of fat mass, the results of this study showed that the InBody 720 estimation of fat mass in elderly people is highly correlated with the DXA measurements (*r* = 0.952). The results of the Bland–Altman consistency analysis show that InBody 720 tends to underestimate fat mass, but the estimations were still consistent overall. This result is the opposite of that in a study in community-dwelling elderly people in Japan, in which Kim et al found that BIA (InBody 720) overestimates whole body fat mass.^[22]^ The possible reason for the inconsistency in the results may be the different degrees of obesity in the subjects. Chen et al studied the body composition of healthy populations in Taiwan and compared the differences in sexes and degrees of obesity to validate BIA (Tanita BC-418) measurement results. The authors’ results show that as the degree of obesity of the subjects increases, the BIA (Tanita BC-418) measurement results may become increasingly inaccurate. In males, lean weight leads to an overestimation of the fat percentage, while normal weight and overweight leads to an underestimation of the fat percentage. In females, lean, normal, and overweight lead to an underestimation of the fat percentage, and the degree of deviation increases as the fat ratio increases.^[24]^ A Canadian study also found that the size and direction of the difference between BIA (Bodystat QuadScan 4000) and DXA measurements is dependent on the fat percentage of the subject. When the subject is overweight, BIA (Bodystat QuadScan 4000) tends to underestimate the amount of body fat. When the subject is thin, BIA (Bodystat QuadScan 4000) tends to overestimate the amount of body fat.^[25]^ Therefore, the degree of obesity of the subject is regarded as an important factor that affects measurement accuracy.

In clinical applications, a new predictive model that is developed based on InBody 720 can be used as an alternative method for estimating body composition in elderly people and may be a simple and highly accurate investigative tool for epidemiological studies. Muscle mass decreases as an individual's age increases. If comorbid reduced muscle strength or physiological abnormalities are present, then this condition is also known as sarcopenia. Sarcopenia can cause disability, reduce one's quality of life, and increase the risk for death in elderly people.^[26]^ If decreased muscle mass below the standard level is the only sign present in elderly people, then this condition is termed presarcopenia. If low muscle mass is accompanied by low muscle strength or low physical function, then this condition is termed sarcopenia. If all three criteria are present, then this condition is termed severe sarcopenia.^[27]^ The common factor for these 3 stages is reduced muscle mass. Therefore, muscle mass is an important indicator for the clinical detection of sarcopenia. Thus, accurate estimations of muscle mass in elderly people is of vital importance. The original InBody 720 measurements will result in an underestimation of muscle mass. However, with the new predictive model, the positive predictive value will be higher than that with the original method, and it will be easier to identify presarcopenia patients in the community, enabling the implementation of early intervention measures.

In this study, not only was the reliability and accuracy of existing equipment evaluated, but a new predictive model for body composition was also developed based on basic demographic characteristics of individuals and impedance analysis data. With linear regression, random forest, SVM, and XGBoost calculations, the prediction statuses of different models were compared, and the fewest parameters were used to achieve the highest accuracy. The new predictive model developed in this study for elderly people aged 65 years and above yields measurements that are more accurate than the raw InBody 720 measurements, and the model is cheaper than DXA. Moreover, our predictive model is more convenient and faster to use, and it can be used as an evaluation tool to promote exercise, healthy diets, weight loss, or other healthcare activities in the community.

Our study has the following limitations:

1. The data for the predictive model was limited by the number of people included in the study. The accuracy of our predictive model may be improved. However, our constructed predictive model exhibited better performance than existing methods.
2. The BIA study data were collected using InBody 720, and other types of BIA devices were not used. However, the criteria for sarcopenia in Asian populations are not limited to the type of machine used for the BIA measurements. Therefore, it can be noted that BIA measurements have a certain reliability and accuracy. In addition, InBody 720 has also been used in many other important studies.
3. The predictive model requires the input of 30 to 40 variables, and some variables need to be converted before they can be inputted into the model. This requirement causes convenience limitations in direct use.

However, if the device manufacturers can program this formula into chips, this limitation can be eliminated.

This study used DXA as the gold standard and employed impedance analysis to develop a new predictive model. This model can prevent systemic errors when BIA is used in measurements in elderly populations. Therefore, this model can be effectively used to evaluate the body composition of elderly people aged 65 years and above. The new predictive model can also be used to monitor the nutrition status of elderly people and identify people with sarcopenia in the community.

## Author contributions

W-HF and S-LS conceived and designed the experiments. W-HF, J-RY, and WS performed the experiments. W-HF, J-RY, and D-JT analyzed the data. W-HF, C-YL, and P-JH provided the reagents, materials, and analysis tools. W-HF, J-RY, and S-LS wrote the paper. M-YT, C-FC, G-SH, and HC were responsible for the critical review and providing comments. S-LS and D-JT were responsible for modifying the manuscript.

Supporting Information: S1 Linear regression formula for muscle mass, fat mass, right arm muscle mass, left arm muscle mass, trunk muscle mass, right leg muscle mass, and left leg muscle mass.

## Supplementary Material

Supplemental Digital Content
