# Supplementary material for: Accuracy augmentation of body composition measurement by bioelectrical impedance analyzer in elderly population
Source: Medicine (Baltimore). 2020 Feb 14;99(7):e19103. doi: 10.1097/MD.0000000000019103 (PMC7035056; doi:10.1097/MD.0000000000019103)
Supplement: Supplemental Digital Content [file medi-99-e19103-s001.docx]

**S1 Linear regression formula for muscle mass, fat mass, right arm muscle mass, left arm muscle mass, trunk muscle mass, right leg muscle mass, and left leg muscle mass**

- **fat mass**

=-14.34235902 +0.131044132 * Z_50kHz-LA_+0.20354819 * ZI_1000kHz-RL_

-0.276689838 * ZI_50kHz-RL_-0.116096228 * ZI_250kHz-LL_+0.160987781 * ZI_5kHz-LL_

-0.195943365 * Z_50kHz-RL_+0.213801543 * ZI_5kHz-LA_-0.151234569 * Z_500kHz-LA_

-0.007792641 * ZI_250kHz-TR_+0.355654946 * ZI_50kHz-LA_-0.002139563 * ZI_1000kHz-LL_

+0.001614927 * ZI_1000kHz-TR_+0.00586068 * Z_50kHz-LL_+0.21275752 * Z_250kHz-RL_

-0.008792443 * Z_500kHz-RA_-0.008283745 * ZI_1kHz-LA_-0.32671089 * ZI_250kHz-LA_

+0.011146583 * Z_1000kHz-LA_ + 0.038415873 * ZI_1kHz-RA_-0.749619575 * ZI_250kHz-RA_

+0.02379218 * Z_5kHz-TR_-0.098070595 * ZI_5kHz-RA_+0.038145611 * ZI_1000kHz-LA_

+ 0.037625792 * ZI_1000kHz-RA_-0.224787493 * ZI_500kHz-LA_+0.57994197 * ZI_50kHz-RA_

+0.148997688 * ZI_500kHz-RA_-2.985927456 *男+0.578175366 *BMI

+0.622137096 *weight

- **muscle mass**

=-12.50895228 -0.02241941 *Z_1kHz-RA_-0.016120957 * Z_50kHz-LA_

+0.100826052 * Z_500kHz-RA_+0.016723098 *age-0.001302679 * ZI_1kHz-TR_

+0.100662719 * Z_250kHz-LA_-0.069655541 * Z_1000kHz-LA_-0.093183937 * Z_250kHz-RA_

-0.080500471 * ZI_1kHz-RL_-0.00140459 * ZI_5kHz-TR_-0.18600217 * ZI_5kHz-RL_

-0.178523346 * ZI_1kHz-LL_+0.15544174 *height+0.087136418 * ZI_5kHz-LL_

+2.669287335 *男-0.0011601 * ZI_1000kHz-TR_-0.00941812 * ZI_50kHz-TR_

+0.004988978 * ZI_250kHz-TR_+0.011901237 * ZI_500kHz-TR_-0.138708927 * ZI_50kHz-LL_

+0.103186116 *weight+0.402837919 * ZI_50kHz-RL_-0.01368096 * ZI_250kHz-RL_

-0.079577374 * ZI_250kHz-LL_ +0.333927028 * ZI_500kHz-LL_-0.51445397 * ZI_500kHz-RL_

-0.066413402 * ZI_1000kHz-LL_+0.425109628 * ZI_1000kHz-RL_+0.192656915 * ZI_1kHz-LA_

-0.263662451 * ZI_5kHz-LA_-0.209884804 * ZI_1kHz-RA_-0.213082493 *ZI_1000kHz-LA_

+0.190987036 * ZI_5kHz-RA_-0.005290819 * ZI_500kHz-LA_-0.084441383 * ZI_50kHz-RA_

-0.036216723 * ZI_1000kHz-RA_-0.780609989 *ZI_250kHz-RA_+0.142939316 * ZI_50kHz-LA_

+0.842137955 * ZI_500kHz-RA_+0.296057307 * ZI_250kHz-LA_

- **right arm muscle mass**

=-1.228960823+0.010213063* Z_5kHz-RA_+0.036056592*BMI

+0.001348148*ZI_500kHz-TR_+0.012715438*height-0.000116764* ZI_1000kHz-TR_

-0.024405102*ZI_1kHz-LL_-0.000971159*ZI_250kHz-TR_-0.017733619* Z_1kHz-RA_

+0.053494247*ZI_50kHz-LL_-0.003219052*age-0.00407476* Z_50kHz-LA_

+0.006167885* ZI_5kHz-LL_-0.006460768*weight-0.039025592* ZI_50kHz-RL_

-0.003496025* Z_250kHz-LA_-0.05366673* ZI_500kHz-LL_+0.064961533* ZI_250kHz-RL_

+0.019951221* Z_500kHz-LA_-0.012751903* Z_1000kHz-LA_+0.222967909*男+0.024495651* Z_50kHz-RA_-0.046181873* ZI_500kHz-RL_+0.015679073* ZI_1000kHz-RL_

+0.019565238* ZI_1000kHz-LL_+0.001934438* ZI_250kHz-LL_+0.139868789* Z_500kHz-RA_

-0.145130573* Z_250kHz-RA_-0.008352224* Z_1000kHz-RA_+0.017943951* ZI_1kHz-LA_

-0.038041846* ZI_5kHz-LA_+0.045815323* ZI_5kHz-RA_-0.09626127* ZI_1kHz-RA_

+0.035454691* ZI_500kHz-LA_-0.033596835* ZI_1000kHz-LA_+0.005212791* ZI_50kHz-LA_

+0.007482002* ZI_250kHz-LA_+0.082370892* ZI_50kHz-RA_-0.023936298* ZI_1000kHz-RA_

-0.449190133* ZI_250kHz-RA_+0.440159861* ZI_500kHz-RA_

- **left arm muscle mass**

= -0.452997124-0.000396202* ZI_250kHz-TR_+0.009983145*height

+0.000606818* ZI_500kHz-TR_+0.007377753* Z_1000kHz-RA_+0.047582356* Z_500kHz-RA_

+0.007534775*weight-0.052213801* Z_250kHz-RA_+0.159245416*男

-0.008577279* ZI_500kHz-LL_-0.004545375* Z_50kHz-LA_+0.016930003* Z_500kHz-LA_

+0.007369285* ZI_1000kHz-LL_-0.000800849* ZI_250kHz-LL_-0.0133605* ZI_500kHz-RL_

+0.002229228* Z_250kHz-LA_-0.017050923* Z_1000kHz-LA_+0.000662517* ZI_250kHz-RL_

+ 0.012611515* ZI_1000kHz-RL_-0.00635459* ZI_1kHz-RA_-0.017430174* ZI_1kHz-LA_

+0.001009519* ZI_5kHz-RA_-0.010634717* ZI_5kHz-LA_+0.027145412* ZI_500kHz-LA_

+0.029977221* ZI_1000kHz-RA_-0.053453684* ZI_50kHz-RA_-0.067391009* ZI_250kHz-RA_

-0.045455951* ZI_1000kHz-LA_+0.084047831* ZI_500kHz-RA_+0.016053906* ZI_50kHz-LA_

+0.040029292* ZI_250kHz-LA_

- **trunk muscle mass**

=-2.732399746 -0.059229074 *BMI-0.040277105 * Z_1kHz-RA_-0.074983716 * Z_1000kHz-LA_

+0.104857908 * Z_250kHz-LA_-0.075214831 * Z_500kHz-RA_+0.089636001 * Z_250kHz-RA_

-0.013352167 * Z_50kHz-LA_-0.00317252 * ZI_1kHz-TR_-0.026491116 * Z_1kHz-RL_

-0.242054395 * Z_1kHz-RL_+1.373012504 *女-0.024233583 * ZI_1kHz-LL_

+0.060054949 * ZI_5kHz-LL_+0.009581755 * ZI_5kHz-TR_-0.001074827 * ZI_1000kHz-TR_

+0.09089284 *height-0.061480905 * ZI_5kHz-RL_+0.182604578 * ZI_50kHz-RL_

+0.260807036 * ZI_500kHz-LL_-0.008277773 * ZI_50kHz-TR_+0.001082493 * ZI_250kHz-TR_

+0.005569265 * ZI_500kHz-TR_+0.102480949 *weight-0.253520704 * ZI_500kHz-RL_

-0.06029351 * ZI_1000kHz-LL_+0.023451522 * ZI_250kHz-RL_-0.028348081 * ZI_250kHz-LL_

+0.152067056 * ZI_1000kHz-RL_-0.119255278 * ZI_5kHz-LA_-0.202729714 * ZI_1000kHz-LA_

-0.356207182 * ZI_1kHz-RA_+0.165401489 * ZI_5kHz-RA_-0.010907454 * ZI_500kHz-LA_

-0.041760387 * ZI_1000kHz-RA_+0.092963203 * ZI_1kHz-LA_+0.285350646 * ZI_500kHz-RA_

+0.087369377 * ZI_50kHz-RA_-0.2665587 * ZI_250kHz-RA_-0.013295725 * ZI_50kHz-LA_

+0.371093106 * ZI_250kHz-LA_

- **right leg muscle mass**

=-2.876043741 +0.009133021 * Z_1kHz-RA_+0.026746275 * Z_500kHz-LA_

+0.002118261 *age-0.02280014 * Z_250kHz-LA_-0.014258843 * Z_250kHz-RA_

-0.012234482 * Z_500kHz-RA_-0.00153499 * ZI_5kHz-TR_-0.000236753 * ZI_1kHz-TR_

+0.01639135 * Z_1000kHz-RA_+0.410668992 *男-0.003688895 * ZI_50kHz-TR_

-0.0000781996985502615 * ZI_1000kHz-TR_-0.046129588 * ZI_1kHz-LL_

+0.008709125 *weight+0.002907118 * ZI_500kHz-TR_-0.0442908 * ZI_5kHz-RL_

+0.013642212 *height+0.002278524 * ZI_250kHz-TR_-0.039235624 * ZI_1kHz-RL_

+0.004768945 * ZI_5kHz-LL_-0.024452224 * ZI_500kHz-LL_-0.009419753 * ZI_250kHz-LL_

+0.065962879 * ZI_50kHz-RL_+0.067502665 * ZI_50kHz-LL_+0.003247519 * ZI_1000kHz-LL_

-0.038699641 * ZI_5kHz-LA_-0.100955828 * ZI_500kHz-RL_+0.01292879 * ZI_5kHz-RA_

+0.038526635 * ZI_1kHz-LA_+0.054828184 * ZI_250kHz-RL_+0.046575013 * ZI_500kHz-LA_

-0.000593542 * ZI_1000kHz-LA_+0.077078931 * ZI_1000kHz-RL_

+0.050297793 * ZI_1kHz-RA_+0.016121305 * ZI_50kHz-RA_+0.051059179 * ZI_50kHz-LA_

+0.042888677 * ZI_1000kHz-RA_-0.081651193 * ZI_250kHz-LA_-0.16959251 * ZI_250kHz-RA_

+0.064411211 * ZI_500kHz-RA_

- **left leg muscle mass**

=-3.035285533 +0.001081933 *age+0.000593974 *Z_1kHz-LL_

-0.020438801*Z_250kHz-LA_+0.014323804 *Z_250kHz-TR_+0.021318932 * Z_50kHz-LA_

-0.003017135 * Z_50kHz-RA_+0.000403573 * ZI_1kHz-TR_+0.001861267 * Z_1000kHz-RA_

-0.003390425 * ZI_5kHz-TR_+0.416466801 *男+0.006342174 * ZI_5kHz-LL_

+0.017457162 *height+0.01112331 *weight-0.026326454 * ZI_1kHz-RL_

-0.001104329 * ZI_50kHz-TR_-0.050788651 * ZI_1kHz-LL_

-0.0000118713880467273 * ZI_1000kHz-TR_+0.003268587 * ZI_250kHz-TR_

-0.049976617 * ZI_5kHz-RL_+0.00109224 * ZI_500kHz-TR_+0.042167093 * ZI_50kHz-LL_

+0.116794881 * ZI_50kHz-RL_+0.058020249 * ZI_1kHz-LA_+0.039012677 *ZI_500kHz-LL_

+0.001572521 * ZI_500kHz-LA_-0.021048197 * ZI_250kHz-RL_-0.111945772 * ZI_500kHz-RL_

-0.019835836 * ZI_250kHz-LL_-0.008925958 * ZI_1000kHz-LA_-0.060490261 * ZI_5kHz-LA_

+0.013991496 * ZI_1kHz-RA_+0.014511848 * ZI_5kHz-RA_+0.096692238 * ZI_1000kHz-RL_

-0.009740014 * ZI_1000kHz-LL_-0.098179056 * ZI_250kHz-LA_+0.131878117 *ZI_50kHz-LA_

+0.011938205 * ZI_250kHz-RA_+0.001270838 * ZI_1000kHz-RA_

-0.048356817 * ZI_50kHz-RA_+0.004170467 * ZI_500kHz-RA_
